# Sarcoidosis Mimicking Malignancy: An Elderly Male With Hypercalcemia and Systemic Symptoms

**DOI:** 10.7759/cureus.82649

**Published:** 2025-04-20

**Authors:** Hadiza Ibrahim, Adil Jumani, Thamer Samha, Luaie Idris

**Affiliations:** 1 Internal Medicine, Zayed Military Hospital, Abu Dhabi, ARE; 2 Pulmonology, Zayed Military Hospital, Abu Dhabi, ARE

**Keywords:** b-symptoms, chronic kidney disease (ckd), hypercalcemia, sarcoidosis, severe hypercalcemia

## Abstract

Sarcoidosis is a multi-system granulomatous disease of unknown etiology that can present with various systemic symptoms and laboratory abnormalities, including hypercalcemia. This case report describes a 74-year-old male who presented with hypercalcemia, weight loss, fatigue, and low-grade fevers, initially raising concern for malignancy. A lymph node biopsy revealed non-caseating granulomas consistent with sarcoidosis. Elevated angiotensin-converting enzyme (ACE) levels further supported the diagnosis. This case highlights the importance of considering sarcoidosis in the differential diagnosis of hypercalcemia and systemic symptoms, especially in elderly patients with complex medical histories.

## Introduction

Sarcoidosis is a chronic, multi-system disorder characterized by the formation of non-caseating granulomas in affected organs. Its etiology remains unclear, but it is believed to involve an abnormal immune response to an unknown antigen. The disease commonly affects the lungs and lymphatic system but can involve virtually any organ, leading to a wide spectrum of clinical manifestations. Hypercalcemia is a less common but significant complication of sarcoidosis, resulting from increased production of 1,25-dihydroxyvitamin D by activated macrophages in the granulomas [1]. Early recognition and appropriate management of sarcoidosis are crucial for improving patient outcomes. This case report details the clinical course of an elderly male who presented with hypercalcemia, weight loss, fatigue, and low-grade fevers, initially raising concerns for malignancy, but was ultimately diagnosed with sarcoidosis.

## Case presentation

A 74-year-old male, with a history of chronic kidney disease (CKD), hypertension (HTN), diabetes mellitus (DM), ischemic heart disease (IHD), nephrolithiasis, benign prostatic hyperplasia (BPH), and previous eye surgery, presented to the nephrology clinic for follow-up due to worsening renal function. Three months prior, the patient experienced a persistent cough, generalized fatigue, poor appetite, and on-and-off fevers reaching up to 38°C. His cough was mostly dry, but occasionally, he produced whitish-yellowish sputum without hemoptysis. The patient was hospitalized in another hospital a month prior to this clinic visit for pneumonia, treated with antibiotics, and showed significant improvement. Post-discharge, the cough persisted, and he reported experiencing chills most evenings, relieved by over-the-counter medication. During his admission for pneumonia, he was found to have hypercalcemia (serum calcium of 5 mmol/L) and worsening renal function (eGFR drop from 30 to 15 mL/min/1.73m²), hence the referral. His hypercalcemia likely contributed to worsening renal function on a background of CKD and prior nephrolithiasis.

The review of systems was significant for fatigue, an unintentional weight loss of 10 kg over three months despite improved appetite, occasional mild frontal headaches, but no other symptoms such as chest pain, shortness of breath, palpitations, gastrointestinal or genitourinary complaints, neck pain, eye redness, or skin rash. The patient was on multiple medications, including bisoprolol, aspirin, clopidogrel, sitagliptin, rosuvastatin, alfuzosin, pantoprazole, and sodium bicarbonate to manage his comorbid conditions. He is active and independent in his daily living activities at baseline. He is a lifetime non-smoker. There was no known family history of malignancy, sarcoidosis, or autoimmune disease.

On physical examination, vital signs were stable with mild dehydration noted. HEENT (head, eyes, ears, nose, and throat) examination revealed no erythema or exudates but one palpable lymph node (LN) in the right axilla, approximately 2 x 2 cm, round, soft, non-mobile, and slightly tender. Respiratory examination showed bilateral fine basal crepitations without wheezes or finger clubbing. Cardiovascular examination was unremarkable with a regular rate and rhythm, no murmurs, or edema. Abdominal examination was soft and non-tender with normal bowel sounds, no costovertebral angle (CVS) tenderness elicited.

**Figure 1:**
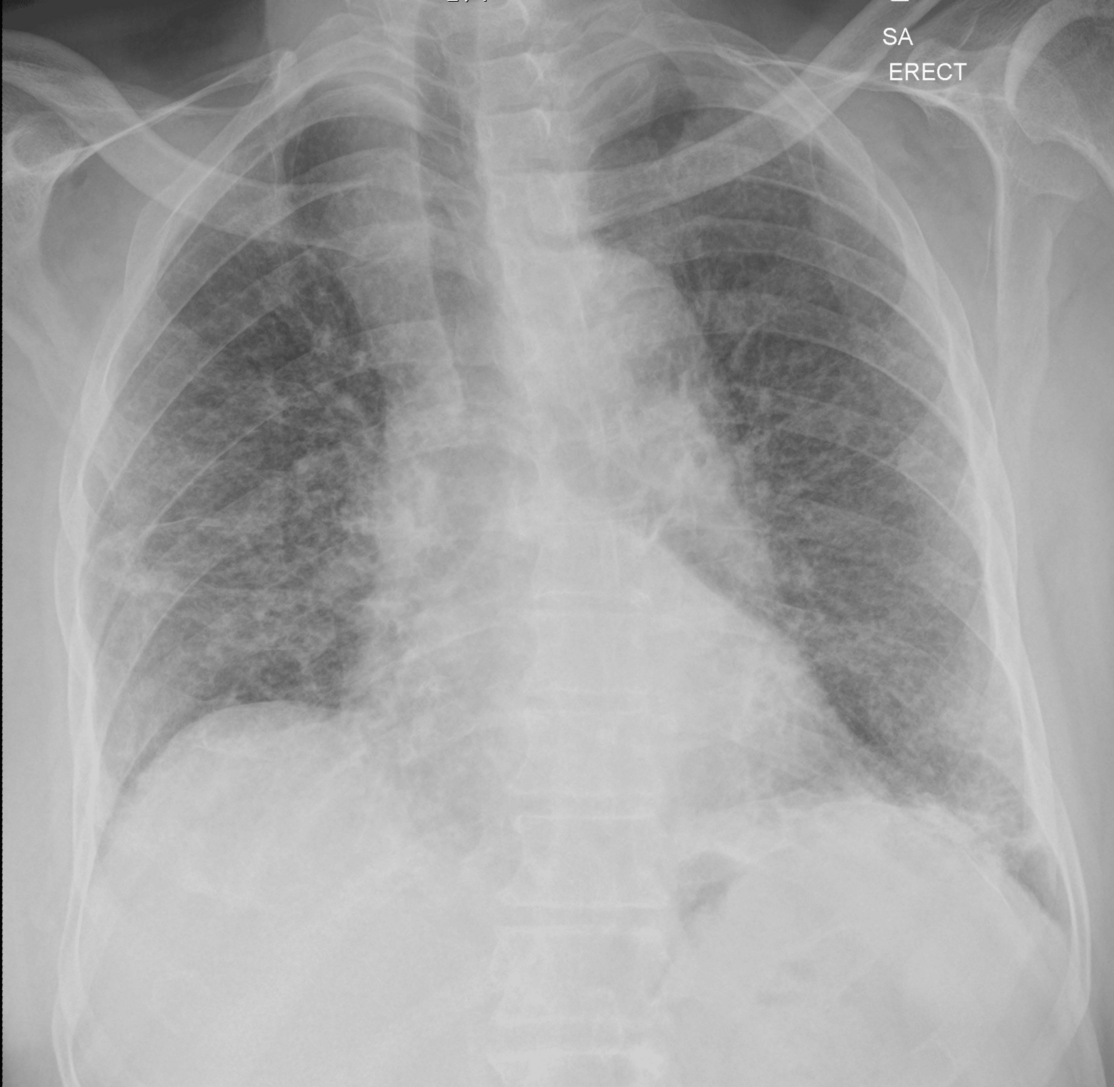
Chest X-ray showing diffuse bilateral fibrotic changes in the lungs

**Figure 2:**
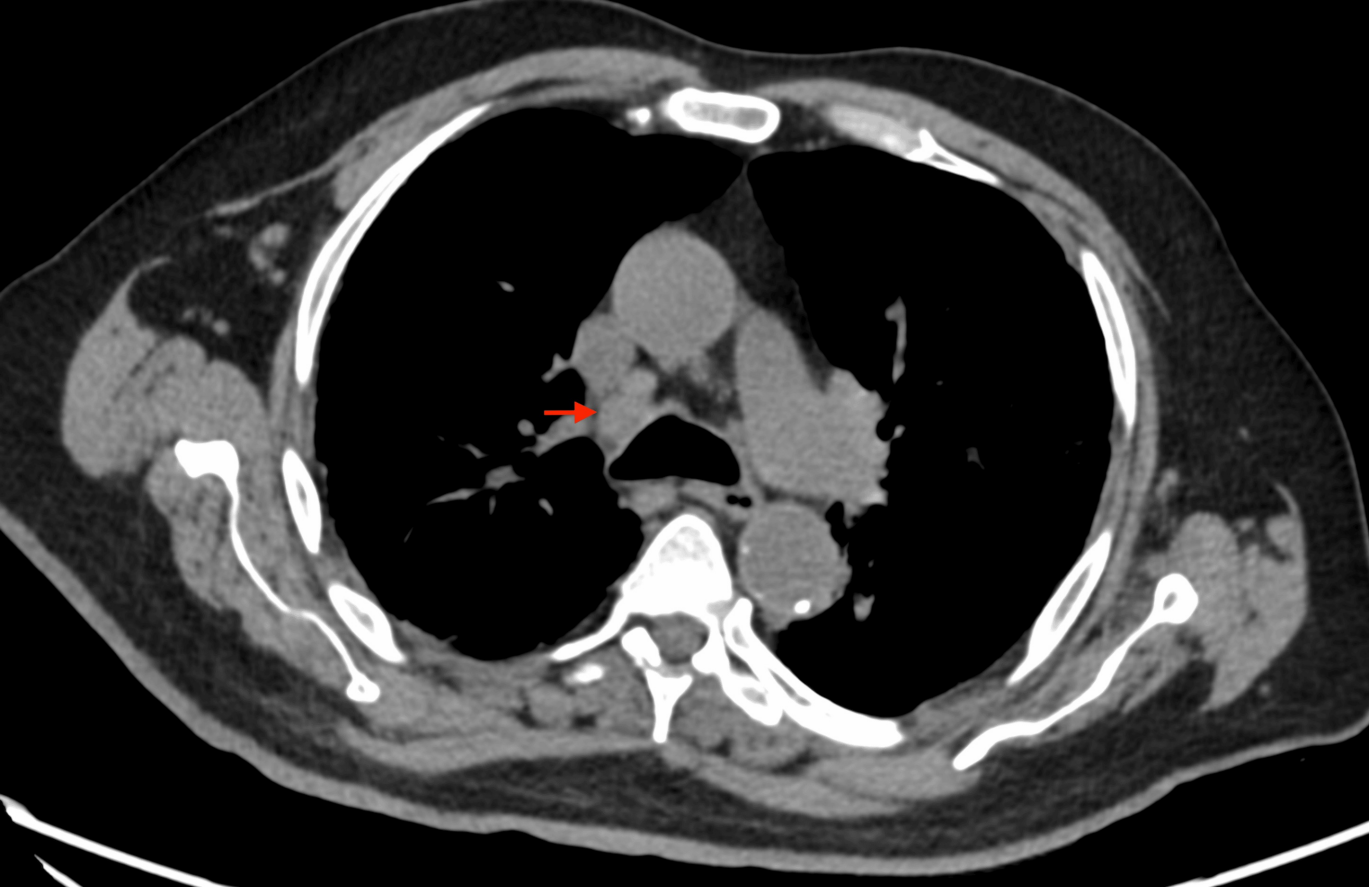
Computed tomography (CT) scan of the chest showing enlarged mediastinal lymph nodes The red arrow represents an enlarged pretracheal lymph node.

**Figure 3:**
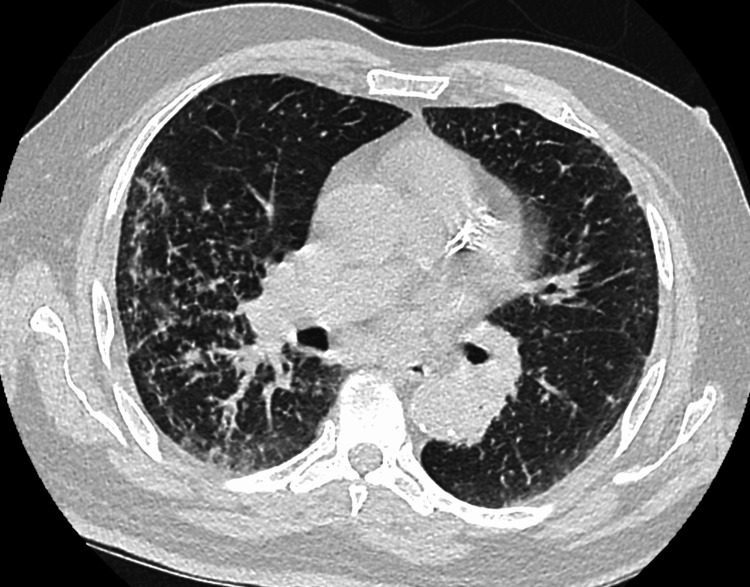
Chest computed tomography (CT) scan showing bilateral fibrotic changes in the lungs and fissure nodularity

Laboratory and imaging studies revealed hypercalcemia with serum calcium of 2.8 mmol/L, low phosphorus (1.38 mmol/L), low parathyroid hormone (PTH of 0.73 pmol/L), low PTH-related peptide, low vitamin D levels, and negative serum and urine protein electrophoresis. Elevated angiotensin-converting enzyme (ACE) levels of 127 nmol/mL/min (normal < 40 nmol/mL/min) were also noted. The electrocardiogram (ECG) was normal, and chest X-ray showed diffuse reticulonodular opacities (Figure 1). Given the findings, the differential diagnosis for non-PTH-mediated hypercalcemia included lymphoma, sarcoidosis, and lung malignancy. The patient was admitted for further workup, including LN biopsy. Computed tomography (CT) scans of the chest (Figures 2-3) revealed lymphadenopathy in the mediastinum, bilateral fibrotic lung changes, and fissure nodularity.

**Figure 4:**
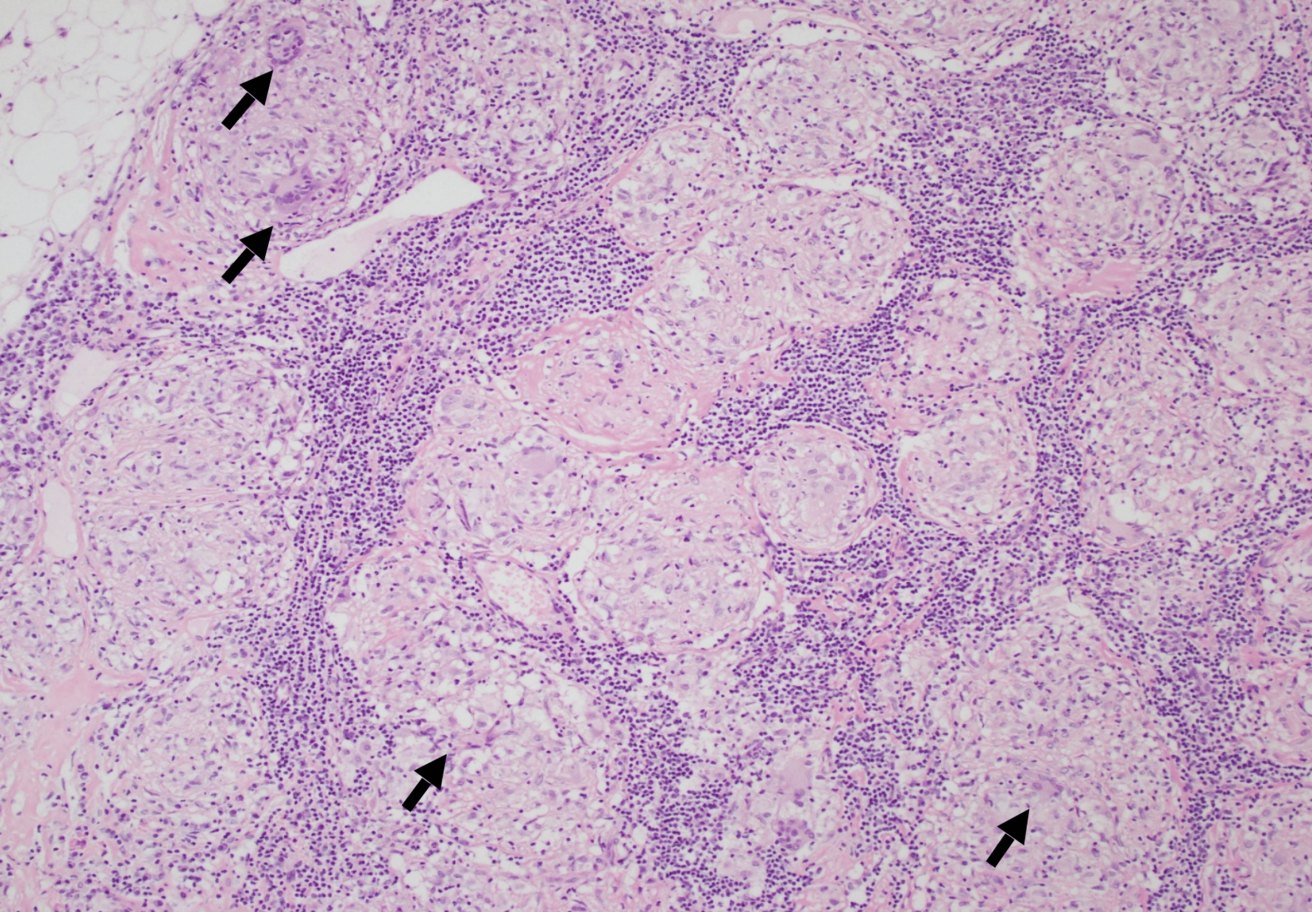
Lymph node tissue studded with multiple granulomas (nodular and fused epithelioid histocytic aggregates) with scattered multinucleated giant cells “arrows”. Note the lymphoid tissue in the background.

**Figure 5:**
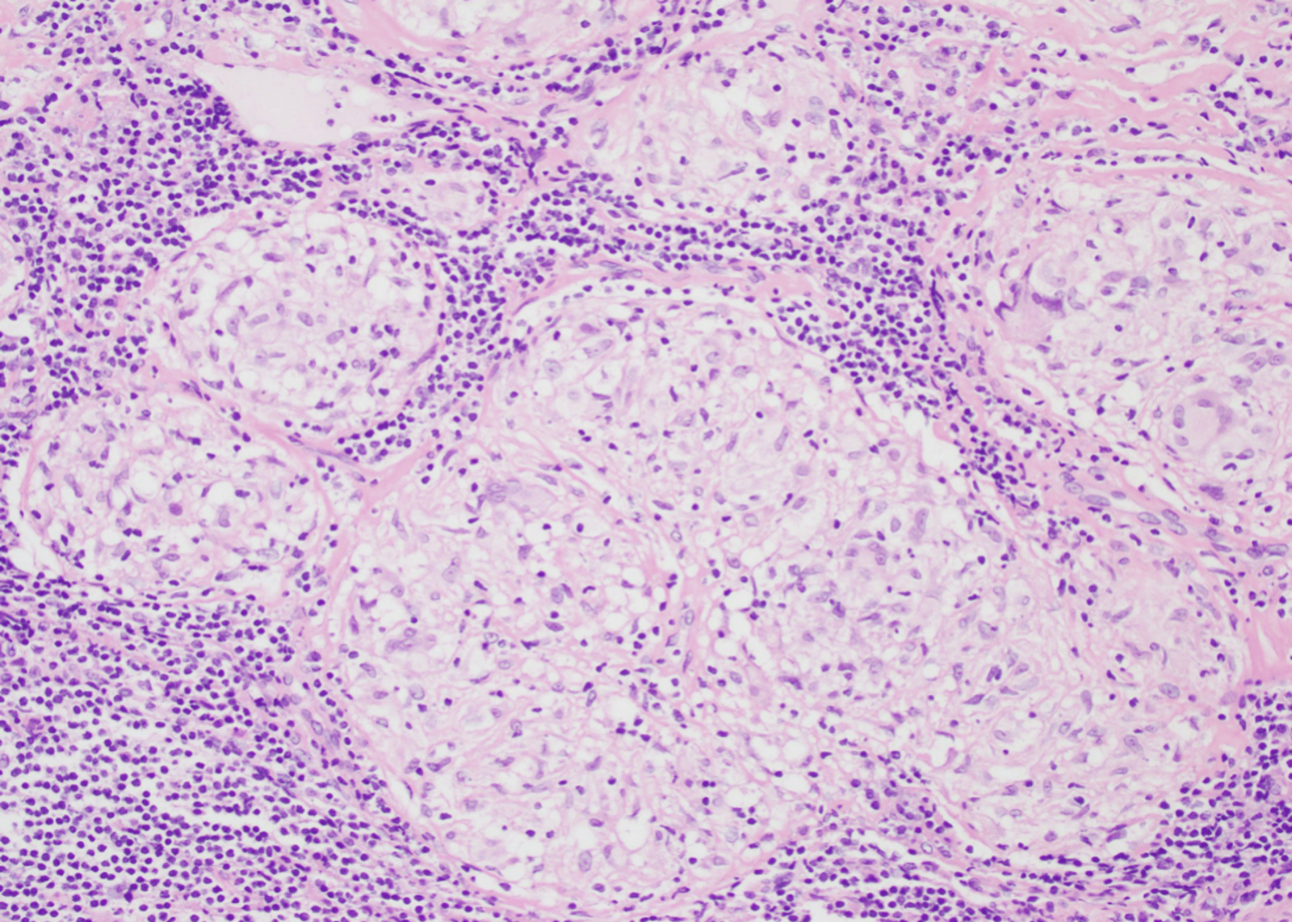
Higher magnification of the lymph node shows multiple granulomas without necrosis (non-necrotizing granulomatous lymphadenitis)

**Figure 6:**
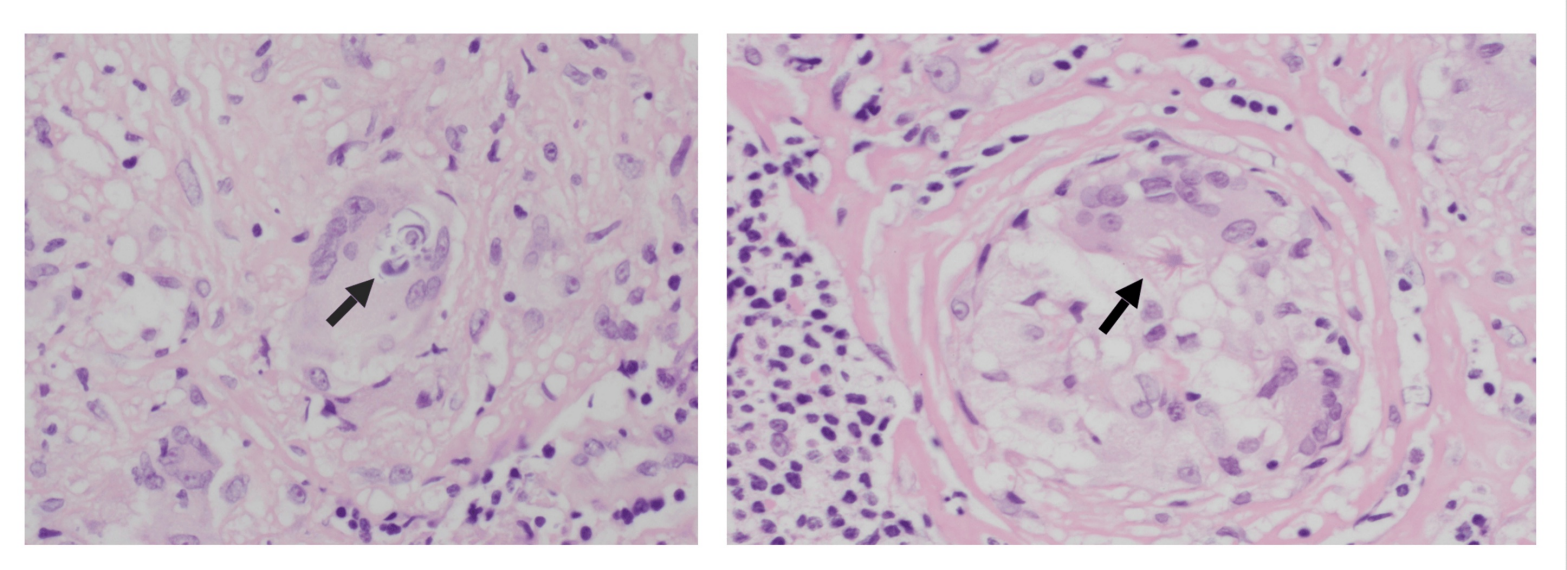
Inclusion bodies can be seen in sarcoidosis (not specific or pathognomonic) noted in the lymph node sections Schaumann body (laminated basophilic calcification = arrow left image) and asteroid body (intra-cytoplasmic eosinophilic star-shaped structure = arrow right image)

During hospitalization, the patient underwent mediastinoscopy with paratracheal and pretracheal LN biopsies and received intravenous normal saline for hypercalcemia (2.94 mmol/L) and other supportive treatment. The LN biopsy (Figures 4-6) revealed non-caseating granulomas, consistent with sarcoidosis, and special stains were negative for Mycobacterium and fungal organisms. Elevated ACE levels further supported the diagnosis of sarcoidosis. He had a low serum lactate dehydrogenase (LDH) of 108 U/L (reference range: 125-220) and a negative QuantiFERON tuberculosis (TB) test. 

The patient was started on oral prednisolone 30 mg daily, with tapering to 20 mg planned over two to four weeks and continuation at that dose until pulmonology review. At follow-up, his symptoms had improved significantly, with no shortness of breath, improved energy levels, no cough, and no fevers.

## Discussion

This case underscores several important clinical aspects, including the wide spectrum of possible presentations of sarcoidosis, particularly in elderly patients with complex medical histories, where the diagnosis may not be considered early. Sarcoidosis is a multi-system granulomatous disorder, predominantly affecting the lungs and lymphatic system but capable of involving any organ system. While pulmonary involvement is most common, extrapulmonary manifestations such as hypercalcemia can be part of the clinical picture [2]. Hypercalcemia occurs in 5-10% of patients with sarcoidosis and is primarily due to increased production of 1,25-dihydroxyvitamin D (calcitriol) by macrophages within the granulomas [1]. This increased calcitriol leads to enhanced intestinal calcium absorption and mobilization of calcium from bones. In this case, the patient's hypercalcemia was significant, with serum calcium reaching 5 mmol/L, necessitating aggressive management, including the use of bisphosphonates (zoledronic acid) and denosumab, an antibody against receptor activator of nuclear factor kappa-Β ligand (RANKL).

The patient’s presentation with non-specific symptoms such as cough, fever, weight loss, and fatigue, coupled with hypercalcemia and lymphadenopathy, initially raised strong suspicions for malignancy, particularly lymphoma. These symptoms, often referred to as "B symptoms" in the context of malignancy, can also be seen in inflammatory and infectious conditions, thereby complicating the diagnostic process [3]. In elderly patients, distinguishing between these entities is particularly challenging due to the prevalence of comorbid conditions and the atypical presentations of diseases. Radiologic findings of diffuse reticulonodular opacities on the chest X-ray and lymphadenopathy on CT scans are non-specific but consistent with sarcoidosis [4]. "Fissure nodularity," seen on CT, is more characteristic of sarcoidosis [5].

The diagnosis of sarcoidosis lacks a standardized protocol and relies on three key criteria: a clinical presentation that fits the disease profile, evidence of nonnecrotizing granulomatous inflammation in tissue samples (though this is not always mandatory), and the exclusion of other potential causes of granulomatous disease [6].

The management of sarcoidosis varies depending on the organ systems involved and the severity of the disease. In this case, the patient's symptomatic hypercalcemia required prompt treatment with IV fluids, bisphosphonates, and RANKL inhibitors. Long-term management often involves corticosteroids to control granulomatous inflammation. For patients who are refractory or intolerant to steroids, alternative agents such as methotrexate, azathioprine, or tumor necrosis factor-alpha (TNF-α) inhibitors may be considered [7].

The patient’s CKD posed additional management challenges. The patient's prior nephrolithiasis may have contributed to renal vulnerability, and hypercalcemia associated with sarcoidosis could have worsened pre-existing renal dysfunction. Hypercalcemia exacerbates renal dysfunction by causing nephrocalcinosis and impairing renal concentrating ability. Careful monitoring of renal function and electrolyte balance is essential in such patients. Additionally, the presence of diabetes and cardiovascular disease complicates the use of certain medications and requires a multidisciplinary approach to optimize overall health outcomes.

The prognosis of sarcoidosis is variable and depends on the extent and severity of organ involvement. While many patients experience spontaneous remission, others may develop chronic disease with significant morbidity. Regular follow-up is necessary to monitor for disease progression, treatment side effects, and complications. Serial imaging, pulmonary function tests, and biochemical monitoring are integral components of follow-up care [8].

## Conclusions

Sarcoidosis can present with hypercalcemia and symptoms that mimic malignancy, making early recognition and comprehensive diagnostic workup essential to avoid unnecessary treatments. This case demonstrates the variable presentations of sarcoidosis and the importance of integrating clinical evaluation, imaging, laboratory investigations, and tissue biopsies. A multidisciplinary approach is important for accurate diagnosis and effective management, ensuring better patient outcomes.
